# Multiple gastrointestinal metastases of squamous-cell lung cancer: A case report

**DOI:** 10.1097/MD.0000000000011027

**Published:** 2018-06-15

**Authors:** Xinyu Li, Songhe Li, Zhiming Ma, Shutao Zhao, Xudong Wang, Dacheng Wen

**Affiliations:** a Department of Gastrointestinal Nutrition and Hernia Surgery, the 2nd Hospital of Jilin University; b Experimental Surgery Division at University of Munich (LMU), Munich, Germany; c Department of Ophthalmology, the 1st Hospital of Jilin University, Changchun, China.

**Keywords:** characteristics, gastrointestinal metastasis, lung cancer, prognosis

## Abstract

**Rationale:**

Gastrointestinal multiple metastases of lung cancer are extremely rare. The majority of gastrointestinal metastasis cases are diagnosed at a late stage and the prognosis is extremely poor. This report describes the clinical characteristics and outcomes of a patient with gastrointestinal multiple metastases from squamous-cell lung cancer, with special emphasis on the diagnosis and treatment of metastatic lung cancer.

**Patient concerns:**

A 61-year-old man who presented with progressive abdominal distention was admitted to our hospital. Radiological examinations showed changes of post-primary pulmonary tuberculosis and mechanical obstruction of the small bowl. Histopathological findings of gastroscopic examination and biopsy specimens showed a diagnosis of squamous-cell carcinoma in the body of the stomach.

**Diagnoses:**

Postoperative histopathology confirmed a gastrointestinal multiple squamous-cell carcinoma in stomach and small bowl. Finally, squamous-cell lung cancer was confirmed by lung biopsy.

**Interventions:**

During his hospitalization urgent surgery was performed because of acute abdomen. The patient underwent a laparotomy with curative gastrectomy for gastric cancer and small bowel partial resection. The patient was recommended with combination chemotherapy of carboplatin and paclitaxel for 3 cycles.

**Outcomes:**

Six months later after operation, the patient succumbed to respiratory failure.

**Lessons:**

We searched the related literature of gastrointestinal metastases from lung cancer and the clinical presentation, site of metastasis, diagnosis, treatment, and survival time in these cases were reviewed. The present study may increase the awareness of early diagnosis and appropriate treatment of metastatic lung cancer of gastrointestinal tract.

## 1 Introduction

Lung cancer is considered one of the most commonly diagnosed cancers, which ranks the leading cause of cancer death among men and the second leading cause of cancer death among women worldwide.^[1]^ The incidence of lung cancer is high in both developing and developed countries, reflecting a prevalence of risk factors, including smoking and other environmental risk exposures.^[1,2]^ Metastasis is a final stage of tumor progression and the majority of lung cancer patients suffered from metastatic disease at diagnosis. Metastasis through blood vessels is the common route for lung cancer and the liver, adrenal glands, brain, and bones are the most common sites.^[3]^ Gastrointestinal tract is not a common site for metastasis of lung cancer and often goes undiagnosed in the clinical follow up of cancer patients.^[4,5]^ Due to near symptomless progression, the majority of gastrointestinal metastasis cases are diagnosed at a late stage, of which the prognosis is extremely poor.^[6]^ Early diagnosis and treatment of these cases are vital for their improving survival. Moreover, gastrointestinal multiple metastases of lung cancer have rarely been reported.

In the present study, the clinical characteristics and outcomes of a patient with gastrointestinal multiple metastases from squamous-cell lung cancer were reported. In addition, the clinical presentation, site of metastasis, diagnosis, treatment, and survival time in these cases were also reviewed. The present study may increase the awareness of early diagnosis and appropriate treatment of metastatic lung cancer of gastrointestinal tract.

This study was approved by the Ethics Committee of the 2nd Hospital of Jilin University. Written informed consent was obtained from the patient.

## 2 Case report

A 61-year-old man who presented with progressive abdominal distention for 8 days was admitted to a local hospital on Feb 23, 2014. Gastroscopic examination and biopsy specimens were evaluated in the local hospital. Histopathological findings showed a suspected diagnosis of squamous-cell carcinoma in the body of the stomach.

**Figure 1:**
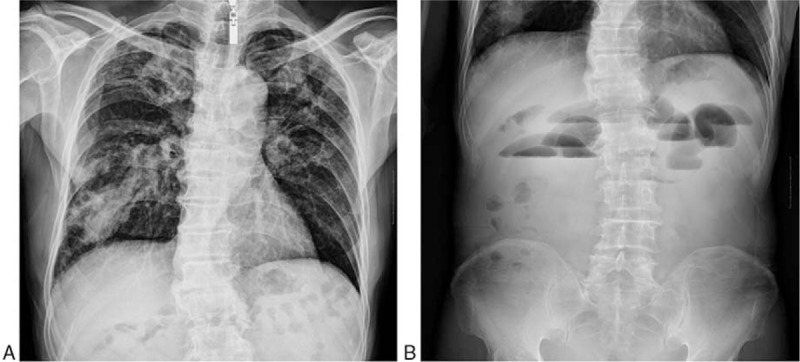
Plain x-rays of chest and abdomen on hospital admission. (A) Chest x-ray showing bilaterally patchy infiltrates, increased bronchovascular markings, and mass in the right lower lung fields. (B) Abdomen x-ray showing showed the gaseous distention of the bowel with air-fluid levels.

**Figure 2:**
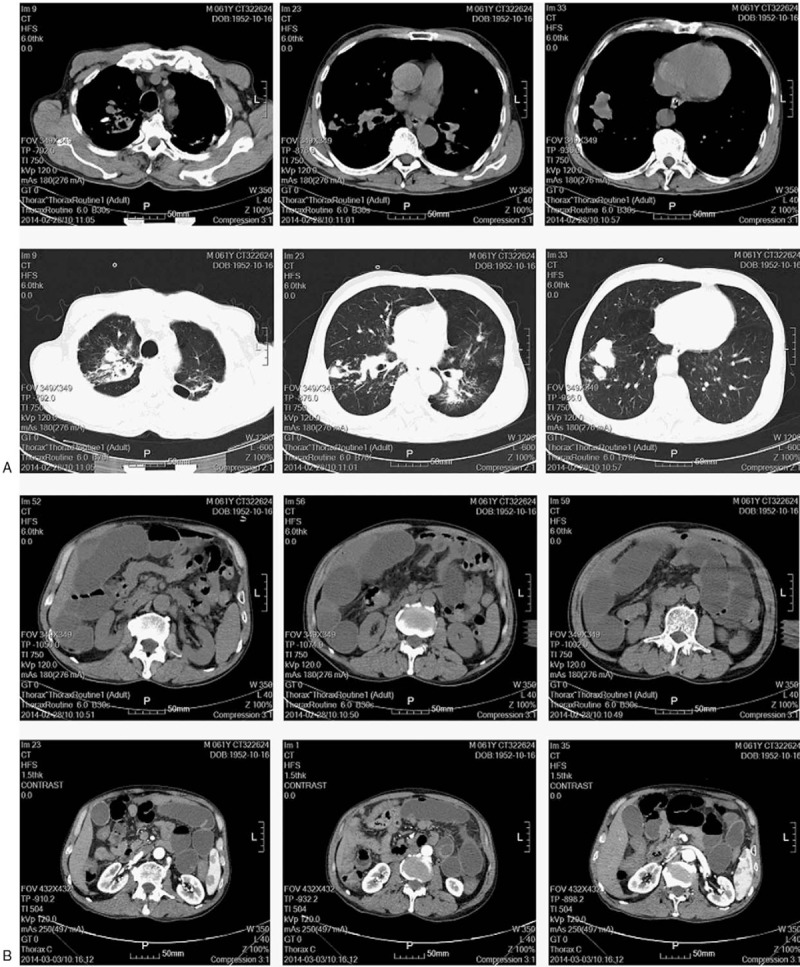
CT scans of chest and abdomen on hospital admission. (A) CT scans of the chest showed bilateral upper-lobe with patchy infiltrates, focal calcification, and nodular opacities peripherally with cavitation. (B) CT scans of the abdomen showed air-fluid levels in loops of small bowel.

**Figure 3:**
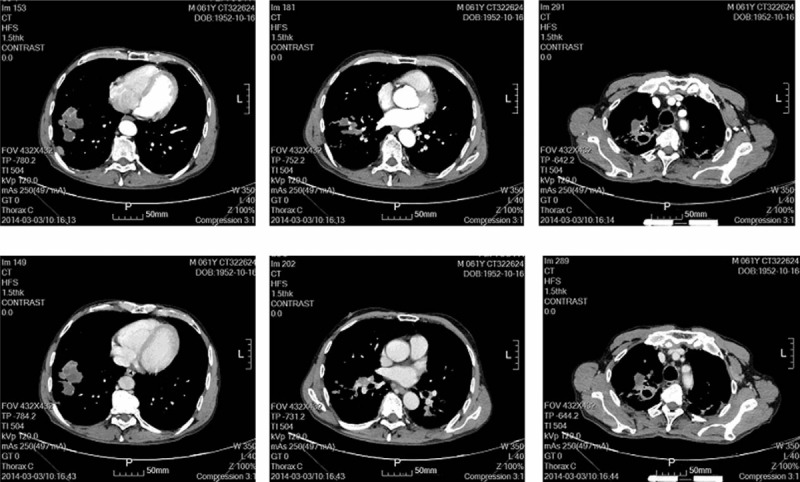
An enhanced CT scan showing that the enhancement of right lower lung fields was not evident.

On Feb 27, 2014, the patient was transferred to our hospital for further management strategy. Physical examination showed that right upper quadrant pain and tenderness were present and no peristaltic waves. The rest of the physical was unremarkable. A detailed medical history was obtained. The patient lost weight of 3 kg in recent 1 month. He had been previously admitted to the hospital for active tuberculosis 5 years ago and finally recovered completely. No history of diabetes, coronary artery diseases, hypertension, hepatitis, drug allergy, previous trauma, or operation was demonstrated. Laboratory tests revealed the following: red blood cell (RBC) count, 4.18 × 10^12^/L (normal range, 3.68–5.13 × 10^12^/L); hemoglobin concentration, 137 g/L (normal range, 114–151 g/L); white blood cell (WBC) count, 11.5 × 10^9^/L (normal range, 4–10 × 10^12^/L); platelet count, 289 × 10^9^/L (normal range, 100–300 × 10^9^/L). Tumor markers were detected as the following: α-fetoprotein (AFP), 3.60 ng/mL (normal range, 0–15 ng/mL); carcinoembryonic antigen (CEA), 1.26 ng/mL (normal range, 0–5 ng/mL); carcinoma antigen (CA) 19-9, 5.6 U/mL (normal range, 50.1–27 U/mL). A chest x-ray showed bilaterally patchy infiltrates, increased bronchovascular markings and mass in the right lower lung fields (Fig. 1A), which needed further computerized tomography (CT) detection suggested by radiologists. A plain x-ray of the abdomen showed the gaseous distention of the bowel with air-fluid levels (Fig. 1B). Abdominal gaseous echoes were detected by ultrasonography. CT scans of the chest showed bilateral upper-lobe with patchy infiltrates, focal calcification and nodular opacities peripherally with cavitation, which indicated the changes of post-primary pulmonary tuberculosis (Fig. 2A); CT scans of the abdomen showed air-fluid levels in loops of small bowel, which indicated that mechanical obstruction was a likely diagnosis (Fig. 2B). Pathologic consultation of gastroscopic biopsy specimens in our hospital confirmed the diagnosis of squamous-cell carcinoma. Given the rarity of gastric squamous-cell carcinoma, pathologists suggested that further examination was needed to find the primary tumor. An enhanced CT scan was performed in this condition and the results were shown that the enhancement of right lower lung fields was not evident, which implied lung sclerosis after tuberculosis (Fig. 3).

**Figure 4:**
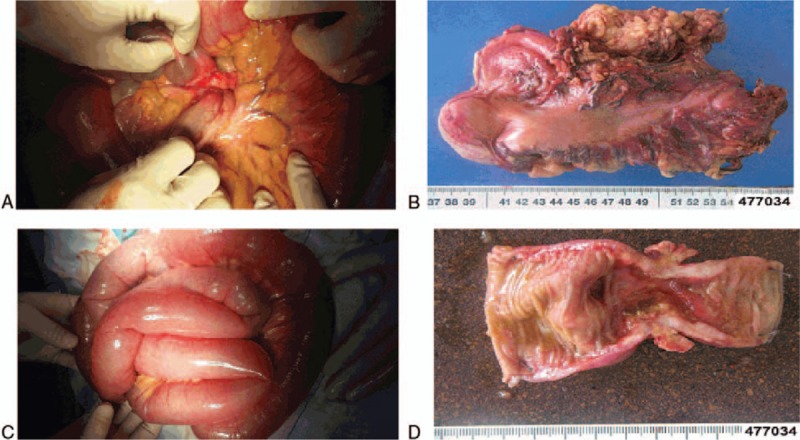
The images of intraoperative view and surgically removed specimens. (A, B) A mass with a diameter of 0.8 cm was palpable in the greater curvature of the stomach. (C, -D) A mass with a diameter of 3 cm was found in the small bowel.

**Figure 5:**
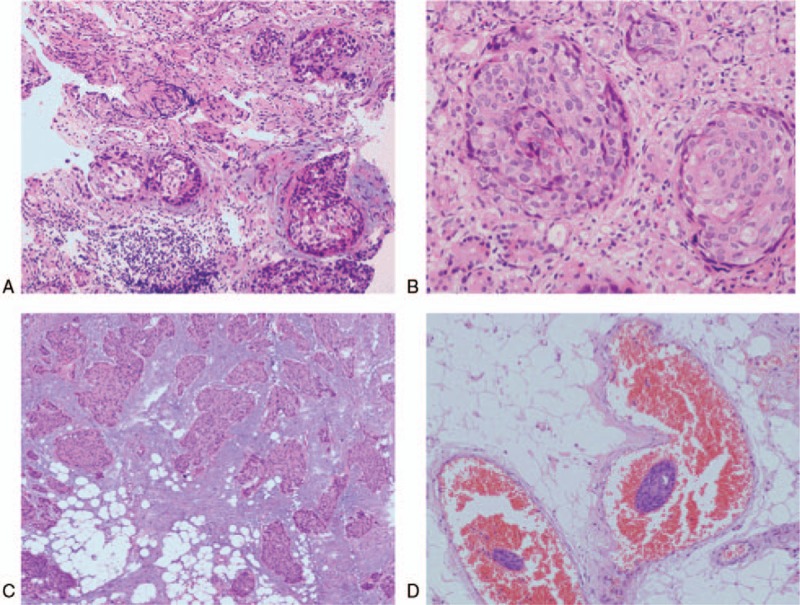
The images of postoperative histopathology. (A, B) Images for gastric slices showing a moderately-differentiated squamous-cell carcinoma. (C, D) Images for slices of small bowl showing a moderately-differentiated squamous-cell carcinoma. A and C were observed under ×200 light microscope; B and D were observed under ×400 light microscope.

On March 5, 2014, the suddenly occurred abdominal pain was persistent and severe. The diagnosis of acute abdomen was made and urgent surgery was needed. The patient underwent a laparotomy with curative gastrectomy for gastric cancer and small bowel partial resection. Intraoperatively, a mass with a diameter of 0.8 cm was palpable in the greater curvature of the stomach and there was no significant lymphadenopathy (Fig. 4A, B). In addition, a mass with a diameter of 3 cm was found in the small bowel at the distance of approximately 30 cm to ileocecal area and the bowel became completely obstructed (Fig. 4C, D). Postoperative histopathology confirmed a moderately-differentiated squamous-cell carcinoma with full-thickness infiltration and vessel invasion in stomach (Fig. 5A, B) and small bowl (Fig. 5C, D). The margins of resection were free of tumor in the specimens and no regional lymph node metastasis was found in perigastric and perienteric area. In view of the rareness of gastrointestinal multiple primary squamous-cell carcinoma, further research was recommended by pathologists to exclude the metastatic squamous-cell carcinoma from the lung or the esophagus.

**Figure 6:**
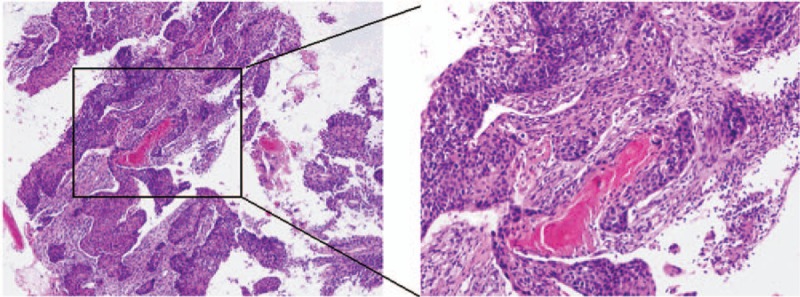
The images of needle biopsy histopathology were shown in (A, B) and squamous-cell lung cancer was confirmed. A was observed under ×200 light microscope; B was observed under ×400 light microscope.

Esophageal mass or lesion was not found on endoscopic examination and the metastatic squamous-cell carcinoma from the esophagus was excluded. It was important to attempt to isolate the tumor cells from the sputum and to carry out a lung biopsy. In the fifth examination of stained smears of sputum, squamous-cells with properties of tumor cells were found. Meanwhile, transbronchial aspirates were obtained with a 19-gauge flexible histology needle by using a CT scan as a guide and squamous-cell lung cancer was confirmed (Fig. 6A, B).

The diagnosis of lung squamous-cell carcinoma with multiple metastases to the stomach and small bowl was confirmed. As the tumor was not resectable, surgeons recommended conservative treatments. The patient was recommended with combination chemotherapy of carboplatin and paclitaxel for 3 cycles. However, in September 2014, the patient succumbed to respiratory failure.

## 3 Discussion

The clinical incidence of gastrointestinal metastasis of lung cancer has been reported to be as low as 0.2% to 1.7%.^[7,8]^ However, autopsy disclosed 4.7% to 14.0% gastrointestinal metastasis of primary lung cancer.^[9,10]^ Most commonly, the clinical course of gastrointestinal metastasis of primary lung cancer is insidious, which make it difficult to diagnose especially in the early stage. In the current study, the patient was diagnosed with gastrointestinal multiple metastases of squamous-cell lung cancer, which has not been reported by other studies. Symptomatic metastatic gastrointestinal lesions from lung cancer are rare and the majority of symptoms caused by metastatic gastrointestinal carcinoma are not noticeable, including weight loss, abdominal pain, meteorism, and constipation.^[11,12]^ The clinical diagnosis of metastatic gastrointestinal tumors is difficult. Gastrointestinal endoscopy and histopathologic diagnosis are the gold standard for metastatic gastrointestinal tumors. Some clinical studies have shown that adenocarcinoma of lung cancer is more likely to metastasize to gastrointestinal tract.^[9]^ In the current study, doctors firstly found the metastatic tumor in gastrointestinal tract and finally confirmed the primary lung squamous-cell carcinoma. Prior tuberculosis increased the difficulty of clinical diagnostic procedures of lung cancer in this patient.

Gastrointestinal endoscopy is an accurate method of identifying patients with gastric, duodenal, or colonic metastatic tumors from lung cancer.^[7]^ However, gastrointestinal endoscopy cannot reach small bowl and capsule endoscopy is a good substitute. CT is one of the most commonly offered by clinical doctors as an estimate of metastasis tumors from the lung. Gastrointestinal metastasis from lung cancer should be considered in the differential diagnosis when CT scans depict short segmental bowel-wall thickening or a polypoid mass in the small intestine in combination with regional lymphadenopathy, perforation, or intussusception.^[8]^ In addition, PET-CT scanning has good sensitivity and specificity to detect metastatic tumors, which is recommended in hospitals available.^[13–15]^ Imaging of the lung is critical for diagnostic decision-making and radiographic characteristics in this patient were masked by sclerosis after tuberculosis.

Histological type of gastrointestinal metastatic lung cancer was squamous-cell carcinoma in the current study, and some clinical studies have demonstrated that squamous-cell carcinoma, large cell carcinoma, and pleomorphic carcinoma of lung cancer are more frequent to metastasize to gastrointestinal tracts.^[16,17]^ However, other certain studies and autopsy series have shown adenocarcinoma to be prominent.^[18,19]^ Therefore, the histological type predominantly associated with gastrointestinal metastasis remains incompletely understood. Immunohistochemical techniques provide an improved method to distinction of the primary source of gastrointestinal metastatic tumors. Immunostaining with TTF-1, CDX2, CK7, and CK20 is helpful in highlighting lung primary.^[11,20]^

The method underlying the spread of metastasis from lung cancer to the intra-abdominal region is believed to involve hematogenous and lymphatic routes.^[21,22]^ The dissemination of cancer cells from the lung through the blood to new organ sites. However, seedling metastasis cannot be ignored in the process of gastrointestinal metastasis. Tumor cells may be swallowed through sputum and induce gastrointestinal seeding. An important future step is needed to clarify the mechanistic details that tumor cells tolerate the nonimmune defense gastric acidity. In the present study, the patient had gastrointestinal multiple metastases of lung cancer and squamous-cells with properties of tumor cells were found in the examination of stained smears of sputum, which may indicate the sputum seedling metastasis hypothesis. However, the mechanism of such hypothesis still remains to be investigated.

Most lung cancer patients with gastrointestinal metastasis exhibit bowel perforation or an acute abdomen. Surgical management should be considered as palliative treatment in patients with bowel obstruction or peritonitis caused by primary lung cancer.^[7,10]^ Postoperative chemotherapy and individualized treatment may improve the survival rate for these lung cancer patients. Some studies have shown that there was no significant difference in recurrence-free survival between patients with metastasis treated with postoperative adjuvant chemotherapy and chemoradiotherapy.^[23]^ In addition, vascular-targeted therapy has gradually been accepted in recent years. Vascular endothelial growth factor-positive patients may benefit more from bevacizumab treatment.^[24]^ Previous studies have shown that the time interval between diagnosis of the primary tumor and manifestation of gastrointestinal metastasis ranged between 2 week and 4 years and the mean time between the identification of the gastrointestinal metastasis and mortality was 100.6 days.^[18,25]^ Because of the poor survival of the majority of patients, the early diagnosis and the development of novel systemic therapies for gastrointestinal metastasis of lung cancer are of paramount importance.

In conclusion, gastrointestinal multiple metastases of lung cancer are extremely rare. The majority of gastrointestinal metastasis cases are diagnosed at a late stage. Despite newer methodology in treatment of this condition, the prognosis still remains poor. Therefore, the presence of clinical gastrointestinal metastasis may be life threatening and comprehensive evaluations are required to detect and monitor gastrointestinal metastasis during follow-up.

## Author contributions

**Conceptualization:** Dacheng Wen.

**Data curation:** Shutao Zhao.

**Investigation:** Songhe Li.

**Methodology:** Songhe Li.

**Resources:** Zhiming Ma.

**Software:** Shutao Zhao.

**Validation:** Xudong Wang.

**Visualization:** Zhiming Ma.

**Writing – original draft:** Xiyu Li.

**Writing – review and editing:** Dacheng Wen.
